# Distributive fairness during the transition to adolescence: The role of peer comparison and social value orientation

**DOI:** 10.1002/pchj.800

**Published:** 2024-09-18

**Authors:** Siqi Liu, Xinmu Hu, Weijun Ge, Xiaoqin Mai

**Affiliations:** ^1^ Department of Psychology Renmin University of China Beijing China; ^2^ Laboratory of Department of Psychology Renmin University of China Beijing China

**Keywords:** adolescents, children, fairness, peer comparison, social value orientation

## Abstract

Combining the dictator game (DG) and the ultimatum game (UG), this study recruited 546 Chinese children (321 boys, aged 9–12 years) as distributors, and found that both peer comparison and social value orientation (SVO) significantly influenced children's distributive fairness from late childhood to early adolescence. Results showed that as the unfairness of peer proposals increased, participants decreased the amount of gold coins distributed to the receiver in both tasks, revealing a peer comparison effect. This effect was more pronounced for adolescents than for children in both tasks. In addition, participants' fair distribution behaviors in the DG showed a three‐way interaction effect of SVO, grade, and peer comparison. Specifically, for proselfs, children were not influenced by peers and consistently proposed self‐interested distributions, whereas adolescents exhibited a peer comparison effect; for prosocials, both children and adolescents were influenced by peers, but children decreased the amount of their distributions only when they saw peers make extremely unfair distributions, whereas adolescents decreased the amount of their distributions when they saw peers make both mildly and extremely unfair distributions. This study highlights the importance of social environment and personal trait in shaping children's fair distribution behavior during the transition from late childhood to early adolescence.

## INTRODUCTION

Children undergo their first considerable shift in social identity upon entering elementary school. Rapid societal expansions and complex interpersonal situations compel children to distribute resources in accordance with social norms, particularly “fairness” (Hashimoto & Toda, 2019; Hsu et al., 2008). The ability of children to achieve distributive fairness serves as a crucial benchmark for their moral cognition and forms the foundation for developing various prosocial behaviors (Krettenauer et al., 2019). As children enter early adolescence, they experience another dramatic change. Alongside biological maturity, adolescents' perceptions of fairness evolve from all‐encompassing equalitarianism to equitarianism (Piaget, 1997). Concurrently, their focus on social interaction shifts toward peers. Internal and external changes inevitably lead to the development of fair behavior. The present study investigates how fair distribution behaviors change during the transition from late childhood to early adolescence, and whether such behavioral changes are influenced by both internal individual trait (social value orientation; SVO) and external socio‐environmental factors (peer comparison). This study aims to enhance our understanding of the developmental trajectories and influential factors of individuals' fair distribution behaviors.

### Development of distributive fairness among children

How individuals perceive and exhibit fairness depends on their development stages (Smith et al., 2013). Previous studies have shown that infants possess a nascent perception of fairness, often providing positive emotional feedback to fair individuals (Lucca et al., 2020) and allocating more attentional resources to fair events (DesChamps et al., 2016). Subsequently, individuals progressively internalize the three fundamental principles of fair distribution: equality (equal distribution), contribution (work‐based distribution), and need (need‐based distribution) (Hsu et al., 2008). Without personal interests at stake, children as young as 3 can distribute resources based on the principles of “equality” and “contribution” (Shaw et al., 2012). However, once personal interests are involved, even 5‐year‐olds actually choose to distribute more resources to themselves, despite a stated preference for fair distribution (Smith et al., 2013). This “knowledge–behavior gap” emerges primarily because young children seek relative advantages and aspire for more than others (Blake et al., 2014). Sheskin et al. (2014) further stated that by age 8–10, children are able to bridge this gap, making fair distributions by considering the needs of others and adhering to fairness norms. Thus, it seems that children encounter a critical turning point in the development of fairness during mid‐childhood, from which they progressively develop a comprehensive perception of fairness and exhibit distribution behaviors consistent with this understanding through late childhood.

The above findings do not indicate that an individual's fair distribution behavior necessarily stabilizes across different contexts after late childhood. Leman et al. (2009) recruited 7‐, 10‐, 11‐, 13‐, and 17‐year‐old participants to engage in a dictator game (DG) and an ultimatum game (UG). In each game, participants were asked to decide, as representatives of a three‐person group, how much money to distribute to themselves and another group. They found no age differences in the UG, but found that adolescents distributed significantly less money to other groups than younger children in the DG. This result shows that while adolescents and children display similar levels of strategic fairness, adolescents exhibit greater self‐interest when others cannot reject their offers. Other studies using the DG have confirmed age‐related increases in concern for self‐interest, with 12‐year‐olds distributing fewer resources to others compared with 8‐ or 9‐year‐olds (Ongley & Malti, 2014; Ruggeri et al., 2018).

However, Güroğlu et al. (2009) drew a different conclusion by employing the DG and the UG. Diverging from the methods of previous studies, the researchers asked participants aged 9, 12, 15, and 18 to select between two proposals to determine their own and another person's payoffs. They found that in the DG, all participants selected the self‐interested proposal, suggesting that adolescents and children exhibit comparable self‐interested preferences. Supporting this, a recent behavioral study found that the amount of resources 8‐ to 12‐year‐olds distributed to another stranger remained stable when the stranger was unable to reject the distribution (Asscheman et al., 2020). Furthermore, in the UG, Güroğlu et al. (2009) found that older adolescents select the altruistic proposal more often than younger children. This result suggests that adolescents might be either more concerned with others' interests or possess more developed strategic fairness than younger children.

One review highlighted that the developmental trends in prosocial behaviors are currently controversial (Malti & Dys, 2018). Despite variations in experimental manipulations and findings, the above studies convey one message that there may be another developmental point between children's and adolescents' fair distribution behaviors. The present study began with this question and thoroughly explored the developmental changes that children's fair distribution behaviors undergo as they transition into adolescence. Based on the developmental characteristics of fairness perceptions (Leman et al., 2009; Piaget, 1997) and current empirical evidence (Li et al., 2016), we defined 9‐ to 10‐year‐olds as being in late childhood and 11‐ to 12‐year‐olds as being in early adolescence. After matching children's biological ages with their school ages, we selected fourth graders as late childhood participants and fifth and sixth graders as early adolescence participants.

Additionally, we investigated other factors that might lead children and adolescents to exhibit inconsistent fair distribution behaviors. Fairness behaviors are shaped by the interaction of personal characteristics, social relationships, and situational information. A previous study pointed out that as children enter adolescence (11 years of age), their cognitive functions significantly improve with the maturation of their thoughts and behaviors (Sutter, 2007). During this transitional period, children encounter increasingly complex social interactions, and their focus on interpersonal relationships gradually shifts to peers. Changes in both internal and external environments inevitably influence children's fair distribution behaviors. The present study focused on the effects of personality trait (SVO) and interpersonal relationship (peer comparison) on children's distribution behavior during the transition from late childhood to early adolescence. In the following text, we introduce the relationship between these two factors and fairness distribution behaviors.

### Peer comparison

When distributing resources, individuals consider not only their own gains or losses but also the benefits to others (Camerer, 2003). When there was only one distributor and one receiver, the proposal of fair distribution rested on a game between the social comparison motivation and the fairness motivation (Sheskin et al., 2014). Specifically, individuals with a strong fairness motivation preferred to distribute resources equally, whereas those with a strong social comparison motivation preferred to maximize self‐interests (Güroğlu et al., 2009). However, fair distribution is not always confined to a vacuum binary “distributor–receiver” structure; some researchers have suggested that participants, whether acting as distributors or receivers, might adjust their fairness behaviors based on the decisions of others in similar roles (Chiang & Wu, 2015; Gächter et al., 2013; Ho & Su, 2009). This aligns with social comparison theory, which posits that in the absence of clear standards, individuals are more inclined to compare themselves with similar others in order to obtain a relatively accurate self‐evaluation (Festinger, 1954). Previous studies refer to others who were in the same experimental situation and had the same identity as the participants as their peers, and refer to the phenomenon of peers' fairness decisions shaping participants' fairness behavior as the peer effect (Ho & Su, 2009; Knoll et al., 2015).

Peer effects manifest among both distributors and receivers. For example, a recent economic study found that retailers' perceptions of fairness and their distribution behaviors are significantly influenced by the proposals of other retailers (Du et al., 2018). Ho and Su (2009) recruited participants as receivers in an UG, where they observed other receivers' decisions before deciding to accept or reject the distributor's proposal. The researchers found that peer decisions significantly influenced the participants' acceptance rates. Additionally, they identified that distributive fairness consists of distributional fairness (relative to another game player) and peer‐induced fairness (relative to peers). They further demonstrated that the influence of peers on participants' acceptance behaviors was twice as significant as that of another player. While current research has confirmed the existence of peer effects, there is still a lack of evidence on how children, particularly those transitioning from late childhood to early adolescence, are influenced by their peers when acting as distributors. Moreover, it remains unclear whether children alter their distribution behavior in response to increased unfairness in their peers' proposals. If they do change, we then assume that a peer comparison effect occurs.

Despite the lack of direct evidence, the mechanism by which peer comparison influences children's fair distribution behaviors can be inferred from existing research on adults. Some researchers suggested that adults exhibit behaviors consistent with their peers primarily to significantly reduce discrepancies in outcomes or social status between themselves and their peers (Riyanto & Zhang, 2014). For example, when peers make prosocial decisions, adults correspondingly increase their prosocial behaviors and decrease antisocial behaviors (Fabbri & Carbonara, 2017). However, other researchers argued that peer comparison merely affects fairness cognition among adults without necessarily changing their actual distribution behaviors (Gächter et al., 2017). Even if the participants adjust their behaviors to align with their peers, this could only occur in a situation where their peers' proposals do not harm the participant's self‐interests (Thöni & Gächter, 2015). We believe that the present study can shed light on clarifying the mechanism by which peer comparisons shape children's fair distribution behaviors.

As children transit from late childhood to early adolescence, their interpersonal focus shifts from family to peer groups, and their behavioral references move from parents to peers. This phenomenon is supported by empirical research. For example, Bishop and Beckman (1971) found that, among children aged 9–15 years, self‐reported consistency with peers significantly increased with age, while it significantly decreased with parents. Similarly, Ruggeri et al. (2018) observed that younger children were more inclined to accept distribution advice from adults, whereas adolescents showed a preference for advice from their peers. The aforementioned studies have either shown that peer effects on fair behavior exist in adults, or that peer effects are stronger in adolescents than in children. However, evidence remains limited on whether peers influence children's and adolescents' fair distribution behaviors, and whether this influence changes developmentally across the two stages. In addition, we wondered whether and how these effects change when peer influence increases. Based on previous findings, we hypothesized that adolescents in this study are more likely than children to adhere to their peers' distribution proposals.

### Social value orientation

SVO reflects the degree to which individuals consider the interests of others and adhere to fairness norms, which are the foundation of fair distribution (Hu & Mai, 2021). Individuals with different SVOs display divergent preferences in resource distribution for themselves and others within the contexts of social interdependence. Proselfs adhere to the norm of self‐interest maximization, whereas prosocials adhere to fairness norms, aiming either to distribute benefits equitably or to maximize shared interests (Bono et al., 2020). Some researchers have further argued that the primary goal of prosocial distributors is to ensure outcome equality rather than to maximize shared interests (Zhang et al., 2017). Given that distributive fairness is based on a game between fairness motivation and social comparison motivation, the behavioral differences between prosocials and proselfs may stem from the different weights they attributed to fairness norms (Bieleke et al., 2017).

Unlike external influencing factors such as peer comparison, SVOs shape distribution behaviors based on individuals' inherent different distributional orientations. Specifically, prosocials typically propose fair distributions and accept fewer unfair distributions compared with proselfs (Anderson & Patterson, 2008). In addition, individuals with different SVOs may process peer information in different ways. Previous studies have shown that adults expect others to have the same SVO as themselves, and if others do, such adults learn others' behaviors (Iedema & Poppe, 1994). Similarly, a recent meta‐analysis found that prosocials expect high prosociality in others, which in turn enhances their own prosocial behaviors, while proselfs exhibit an opposite tendency (Pletzer et al., 2018). Wei et al. (2016) examined the effect of group influence on the distribution behaviors among adults with different SVOs, and found that prosocials only followed the prosocial behaviors of group members, whereas proselfs followed all behaviors of group members. Existing research evidence suggests that prosocials are less likely than proselfs to follow peers' unfair behaviors. This view remains to be validated, and more research is needed to explore how prosocial and proself participants adjust their distributive behaviors after seeing peer proposals with varying degrees of unfairness, particularly when the participants are children and adolescents.

Adults' SVO remains stable across time and contexts (Hu & Mai, 2021), and it can be expressed automatically to influence fairness behaviors directly (Cornelissen et al., 2011). However, Li et al. (2013) found that children's SVO can only be stably expressed through behavior after the age of 14. In their study, children aged 9, 11, and 14, along with adults, were asked to distribute money between themselves and another player, both in a hypothetical scenario and in a real payment context. For instance, participants could choose between Option A (4, 7) and Option B (10, 5), where the first number represents the amount distributed to the participant and the second to the other player. In the hypothetical context, participants were asked to imagine making a distribution, and all participants received the same payoff regardless of their imagined choice. In the real payment context, participants' choices determined their real payoffs. The authors argued that this manipulation effectively measured both the intrinsic distributive orientation and the actual distributive behavior. If the percentage of different SVOs changed between the two contexts, it indicated that the expression of SVOs is context‐dependent. The results revealed that 9‐ and 11‐year‐olds had prosocial orientation but exhibited proself behavior, whereas 14‐year‐olds showed cross‐situational consistency in their internal orientation and external behavior, nearly reaching adults' level. This consistency might partially explain why a significantly larger proportion of prosocials is observed in the adult population in the real world. Because the participants in this study were at different developmental stages, we recorded the percentages of prosocials and proselfs across ages to validate the stage‐expressive property of SVOs. If this property does exist, we then hypothesize that there will be significant differences in fair distribution behavior of the two SVOs in late childhood and early adolescence.

### The present study

The DG and the UG are the most frequently used paradigms for investigating the development of fairness. In both games, roles are divided into a distributor and a receiver. Participants always act as distributors to provide distribution proposals; in the DG, the receiver can only accept these proposals, whereas in the UG, the receiver may either accept or reject them. Previous researchers agreed that the DG measures “pure fairness,” whereas the UG measures “strategic fairness” (Bono et al., 2020; Güroğlu et al., 2009). This can be explained as follows: First, in the DG, the distributor considers only personal preferences, whereas in the UG, the distributor must account for the risk of proposal rejection by the receiver (Hu & Mai, 2021). Second, the distributor has absolute power in the DG, but this power is diminished in the UG by the receiver's right to reject proposals (van Dijk & Vermunt, 2000). The concerns for others' interests and reduced power compel distributors in the UG to adjust their distribution strategies to satisfy the receivers. In other words, individuals distribute resources as “independent selves” in the DG and “interdependent selves” in the UG. Combining these two experimental paradigms can help us investigate the development of distributive fairness among children in a significantly comprehensive manner.

In this study, we adapted the DG and the UG to investigate the influence of SVOs and peer comparisons on distributive fairness among children transitioning from late childhood to early adolescence. More specifically, participants with different SVOs act as distributors, distributing gold coins for themselves and another unfamiliar student in both tasks. They watched other distributors' proposals before making their own distributions, facilitating the manipulation of peer comparison. Three peer comparison conditions were established based on the fairness levels of other distributors' proposals: peer fair distribution, peer mildly unfair distribution, and peer extremely unfair distribution. Based on previous studies and the purpose of this study, the following hypotheses were made: (a) Peer comparison would influence participants' fair distribution behavior. Participants would distribute gold coins with reference to the proposals made by peer distributors; the more unfair the proposals made by peer distributors, the fewer gold coins the participants would distribute. (b) The effect of peer comparison would vary at different developmental stages. Given that adolescents are expected to perceive peers as more important than children (Ruggeri et al., 2018), participants in the early adolescent stage would be more likely to follow the proposals of peer distributors than those in the late childhood stage. (c) SVOs would moderate the peer comparison effect. Given that prosocials typically adhere more strictly to fairness norms than proselfs (Wei et al., 2016), we expected that they would be less susceptible to the peer comparison effect on fair distribution behaviors. That is, the number of gold coins distributed by prosocials would not change significantly across different peer comparison conditions.

## METHODS

### Participants

The minimum sample size was calculated using MorePower 6.0.3 (Campbell & Thompson, 2012). For a 2 (SVO) × 3 (Grade) × 3 (Peer comparison) repeated measures analysis of variance (rmANOVA) with SVO and Grade as the between‐subjects factors, a total of 132 participants (22 per group) is required to detect a three‐way within‐between interaction effect with the two‐sided *α* of 0.05, a power of 0.90, and a medium effect size (*η*_p_^2^) of 0.06. After matching the actual age and school age of children at different developmental stages, the present study recruited 546 children in Grades 4, 5, and 6 of an elementary school in China as participants, with Grade 4 participants identified as being in the late childhood stage and Grades 5 and 6 participants identified as being in the early adolescence stage. The details of the participants are shown in Table 1. Informed written consents for participation in this study were obtained from the participants' parents. Oral assents were also obtained from all the children. To compensate the children for their participation in this study, all children received stationery packages of equal value (e.g., pens, notebooks).

**TABLE 1:** Demographic information of participants (*N* = 546).

	Sex		Total	Age (*M* ± SD)
	Male	Female		
Grade 4	111	63	174	10.06 ± 0.48
Grade 5	101	96	197	11.10 ± 0.39
Grade 6	108	67	175	12.12 ± 0.36

### Measurement of social value orientation

Each participant's SVO level was measured using the slider measure (SLM) (Murphy & Ackermann, 2014). Previous studies have demonstrated that the SLM is applicable to a broader age range, and it has better reliability and validity than other instruments (Bieleke et al., 2017). The SLM comprises six questions, each of which requires the participant to choose among nine distribution options. The SVO angle was calculated based on the participant's choice. If the angle was larger than 22.45°, the participant was classified as prosocial; otherwise, they were classified as proself (Murphy & Ackermann, 2014; Pletzer et al., 2018).

### Procedure

After the measurement of SVO, formal experiments were conducted using class units. All participants were provided with an answer booklet to fill out their answers. Next, all participants completed the DG (Task 1) and the UG (Task 2) under the instructions of the experiment.

#### Task 1: The dictator game in peer comparison contexts

The participants were asked to play three rounds of the DG. In each round, the participants (the distributors) had to decide the way in which to distribute 10 gold coins among themselves and a strange student (the receiver) in the same grade and of the same sex, and the receiver could only choose to accept. Unlike the classical studies involving the DG, in this study, we asked the participants to watch another strange distributor's (the peer) proposal before they made the distribution, which enabled the manipulation of peer comparison. Throughout the three rounds of the tasks, three peer distributors chose to distribute seven gold coins (7–3), five gold coins (5–5), or nine gold coins (9–1) to themselves, respectively. It has been demonstrated that those three proposals can adequately represent different levels of fairness (Hu et al., 2021). In other words, each participant in this study watched three peer distribution proposals with different fairness levels: mildly unfair (7–3), fair (5–5), and extremely unfair (9–1) before self‐distribution, and the watching order was fixed as such. The complete procedure of the experiment is presented in Figure S1.

The participants were then asked how many gold coins they wanted to distribute after knowing the other distributor's proposal. Once decided, they had to write down the number on the answer booklet. The participants were also reminded that the coins kept to themselves through three rounds could be exchanged for gifts with different values, and the more the coins, the better the gift. In summary, this task used a mixed design of 2 (SVO: prosocial vs. proself) × 3 (Grade: Grade 4 vs. Grade 5 vs. Grade 6) × 3 (Peer comparison: fair vs. mildly unfair vs. extremely unfair), where “peer comparison” was a within‐participants factor.

#### Task 2: The ultimatum game in peer comparison contexts

The participants were given a 2‐min break before engaging in Task 2, during which no communication was allowed. In this study, each participant was asked to complete the DG task before completing the UG task. The reason for this was that a previous study confirmed that performing the DG first would not affect children's distribution proposals in the UG, whereas performing the UG first would significantly change children's distribution proposals in the following DG (Zhu et al., 2008). Thus, in the present study, the order of presentation of the two tasks was fixed.

The procedure of Task 2 was similar to that of Task 1 (for more details, see Figure S2). Participants were again asked to watch the distribution proposals of the other three peer distributors in the same order before they decided on how to distribute 10 gold coins between themselves and the receiver. However, the receiver in Task 2 could choose to reject the participants' proposals. In Task 2, we again conducted a mixed design of 2 (SVO) × 3 (Grade) × 3 (Peer comparison), and “peer comparison” was a within‐participants factor.

## RESULTS

### Proportion of different social value orientations

**FIGURE 1:**
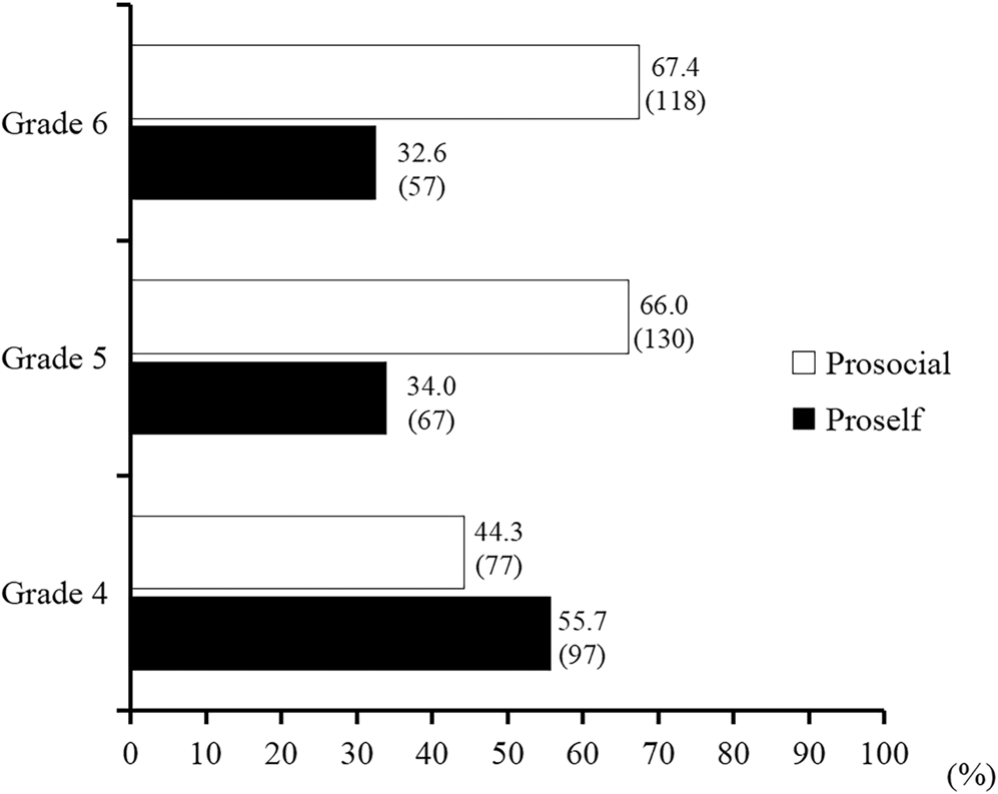
Percentage of different SVOs in Grades 4, 5, and 6. The upper number represents the percentage of prosocials or proselfs in each grade, and the lower number in parentheses represents the actual number of children with different SVOs.

Figure 1 shows the number and percentage of proselfs and prosocials in the three grades. Through a chi‐square test, we found that the proportion of SVOs changed across different grades (*χ*_(2)_^2^ = 24.80, *p* < .001), with a slightly higher proportion of proselfs in Grade 4 and a higher proportion of prosocials in Grades 5 and 6.

### Numbers of distribution

As sex may influence children's distribution behavior, we first included sex as a dependent variable, and performed a 2 (Sex: male vs. female) × 2 (SVO: prosocial vs. proself) × 3 (Grade: Grade 4 vs. Grade 5 vs. Grade 6) × 3 (peer comparison: fair vs. mildly unfair vs. extremely unfair) rmANOVA for both DG and UG using SPSS (version 22.0, SPSS Inc., Chicago, IL, USA) on the number of gold coins distributed by children. The results did not reveal the main effect of sex and the interaction effects including sex in any of the tasks (*p*s > .05); more details can be found in Supplementary Materials. To eliminate possible confounding effects due to differences in sex ratios between grades, we included sex as a control variable and conducted a 2 (SVO) × 3 (Grade) × 3 (Peer comparison) rmANOVA. Table S1 shows the actual number of participants distributed in each grade under three peer comparison conditions.

The main effect of grade occurred only in the UG, *F*(2, 539) = 5.20, *p* = .006, *η*_p_^2^ = 0.02. Post hoc comparisons showed that children in Grade 4 (*M* ± *SD*, 4.97 ± 1.35) distributed more gold coins than children in Grade 5 (4.58 ± 1.40, *p* = .05) and Grade 6 (4.48 ± 1.43, *p* = .007), and no difference was found between Grades 5 and 6 (*p* > .05). In contrast, in the DG, children in all grades distributed comparable amounts of coins (Grade 4: 3.13 ± 1.82; Grade 5: 3.77 ± 1.91; Grade 6: 3.46 ± 1.93), *p*s > .08. The main effect of peer comparison was significant in both the DG and the UG, with the number of children distributed decreasing as the peers' proposals became increasingly unfair, *p*s ≤.001 (see Supplementary Materials Section 2.1). The main effect of SVO was also significant in that the prosocials distributed more coins than the proselfs (DG: 4.06 ± 1.86 vs. 2.96 ± 1.86; UG: 4.95 ± 1.37 vs. 4.39 ± 1.37) regardless of whether it was in the DG or the UG, *p*s < .001 (see Supplementary Materials Section 2.2).

**FIGURE 2:**
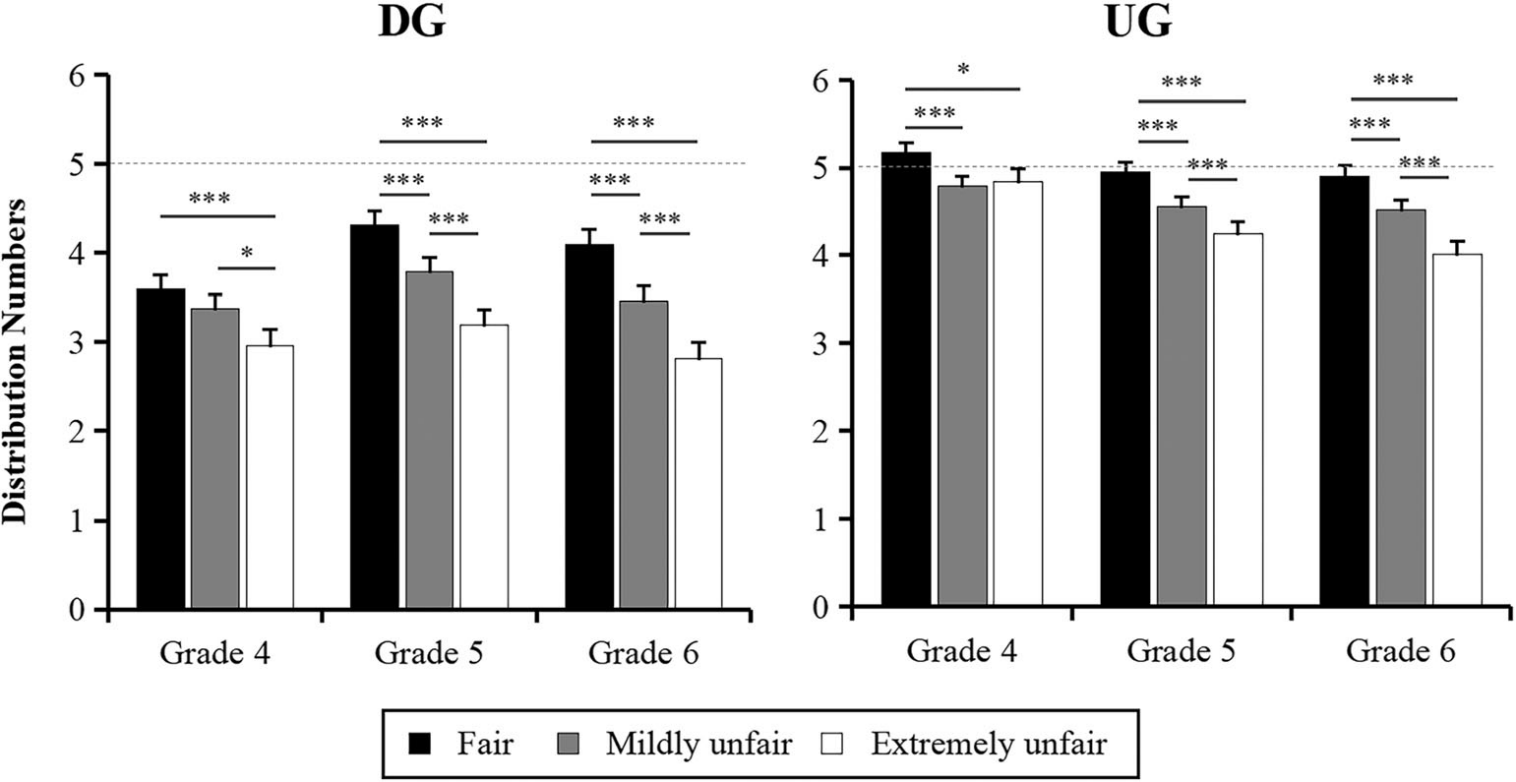
Numbers of distribution in each grade for the DG and the UG under three peer comparison conditions. Error bars represent 1 standard error in each condition. *p < .05, ***p < .001.

Although the interaction between grade and peer comparison was significant in both tasks (DG: *F*(4, 1078) = 2.70, *p* = .032, *η*_p_^2^ = 0.10; UG: *F*(4, 1078) = 3.63, *p* = .008, *η*_p_^2^ = 0.13), the simple effect analysis revealed that as the unfairness of the peer distribution proposal increased, fifth‐ and sixth‐grade children distributed progressively fewer gold coins to others throughout both tasks (*p*s < .022), whereas for fourth‐grade children, the peer comparison effect was different in the DG and the UG. Specifically, in the DG, the distribution number was fewer among fourth‐grade children after viewing the peers' extremely unfair distribution than after viewing the peers' fair distribution (*p* < .001) and mildly unfair distribution (*p* = .011), but there was no difference between the latter two conditions (*p* = .313). However, in the UG, the number of gold coins distributed by fourth‐grade children was significantly fewer in the peers' mildly unfair (*p* = .001) and extremely unfair distributions (*p* = .044) compared with the peers' fair distribution condition, but there was no difference between the first two conditions (*p* > .05) (see Figure 2).

**FIGURE 3:**
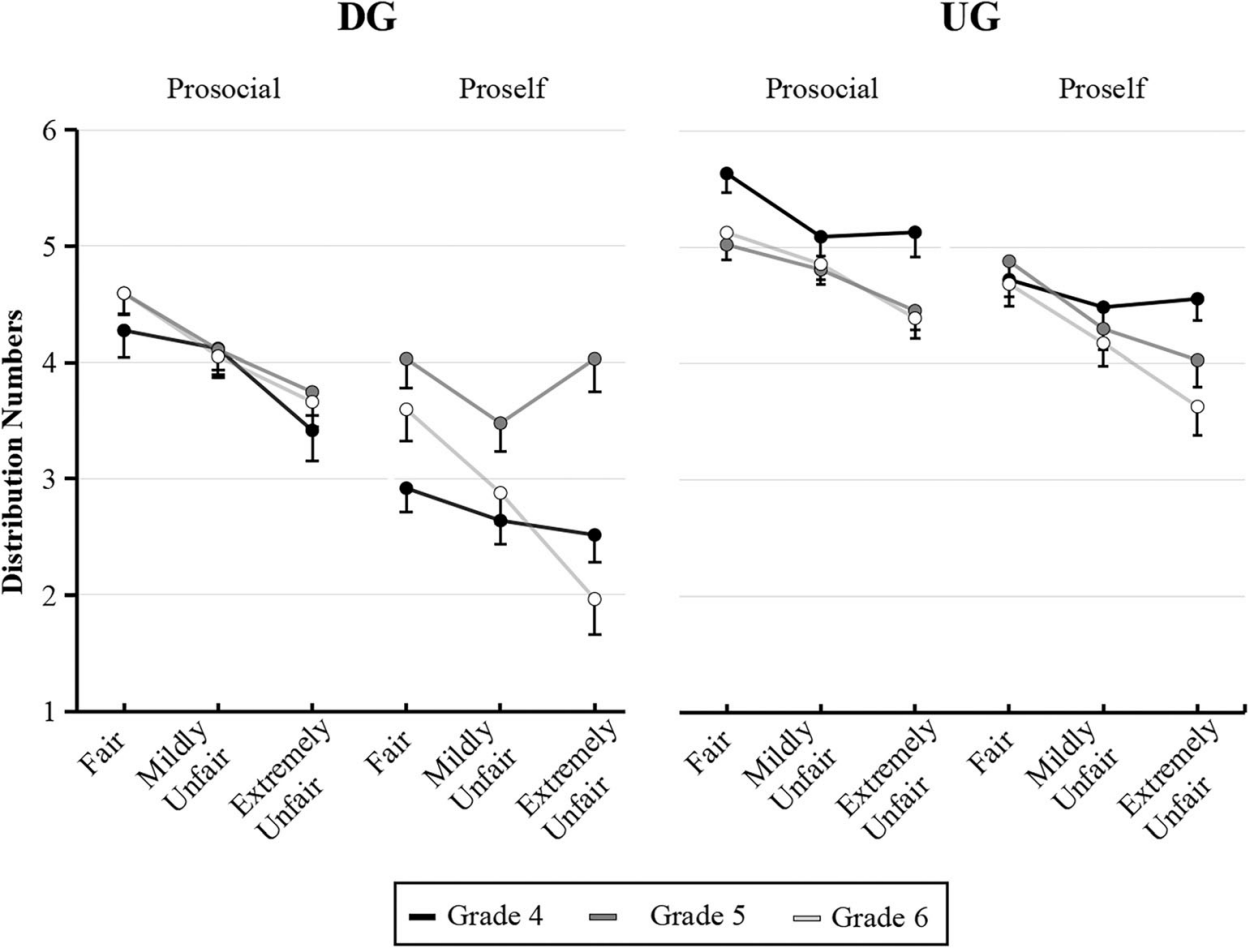
Distribution numbers of prosocials and proselfs under three peer comparison conditions in the DG and the UG. Error bars represent 1 standard error in each condition.

The interaction of SVO × Grade × Peer comparison was significant only in the DG, *F*(4, 1078) = 3.04, *p* = .018, *η*_p_^2^ = 0.01. For participants in Grade 4, there was no significant difference in the number of gold coins distributed to others by the proselfs throughout the three peer comparison conditions (*p*s > .169). However, the prosocials distributed more gold coins to others in the peer fair distribution (*p* = .001) and mildly unfair distribution (*p* = .003) conditions than in the peer extremely unfair distribution condition, and no difference was found between the peer fair distribution and mildly unfair distribution conditions (*p* > .05). For participants in Grades 5 and 6, the number of gold coins distributed by the proselfs reduced considerably as peer distribution unfairness increased (*p*s < .025). However, the prosocials distributed significantly fewer gold coins in both the peer mildly unfair distribution (*p* < .004) and extremely unfair distribution (*p* < .001) conditions compared with the peer fair distribution, and the difference between these two conditions was not statistically significant (*p*s > .07). Although the three‐way interaction was not significant in the UG, further comparative analysis was also conducted, as shown in Supplementary Materials Section 2.3. Figure 3 shows the different distribution trends of participants between the DG and the UG.

### Difference value of distribution numbers between the DG and the UG

To examine the distributional patterns among children more precisely, we computed the difference values (Number_UG_ – Number_DG_) between the UG and the DG for each participant. Then, we conducted a 2 (SVO: prosocial vs. proself) × 3 (Grade: Grade 4 vs. Grade 5 vs. Grade 6) × 3 (Peer comparison: fair vs. mildly unfair vs. extremely unfair) rmANOVA, with sex as the control variable.

**FIGURE 4:**
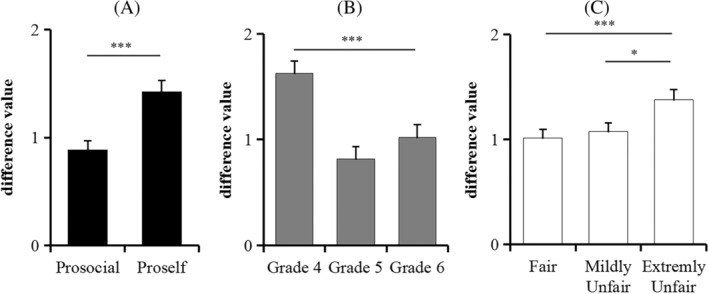
(A) The difference values of the prosocials and proselfs. (B) The difference values in Grades 4, 5, and 6. (C) The difference values in three peer comparison conditions. Error bars represent 1 standard error in each condition. *p < .05, ***p < .001.

The main effect of SVO was significant, *F*(1, 539) = 16.00, *p* < .001, *η*_p_^2^ = 0.03. The difference value was larger for proselfs (1.42 ± 1.49) than for prosocials (0.88 ± 1.44). The main effect of grade was significant, *F*(2, 539) = 12.11, *p* < .001, *η*_p_^2^ = 0.04. The difference value was larger in Grade 4 (1.62 ± 1.54) than in Grade 5 (0.82 ± 1.61) and Grade 6 (1.02 ± 1.64), *p*s ≤ .001, but no difference was found between Grades 5 and 6, *p* = .878. When the difference values were used, the main effect of peer comparison did not reach the significance level, but we found that the difference values tended to increase when peers proposed extremely unfair distributions (1.37 ± 2.34) compared with peers proposing fair distributions (1.01 ± 2.10, *p* = .002) and mildly unfair distributions (1.07 ± 1.87, *p* = .012). None of the interactions were significant (*p*s > .168) (Figure 4).

In addition, we conducted another inter‐task comparison using the actual number of gold coins distributed by participants. The two analyses yielded essentially the same main results. In order to more visually identify the difference between the two tasks, we chose to present the results of the difference values in the main text, and the results of the raw values in Section 4 of Supplementary Materials.

## DISCUSSION

Using the DG and the UG, we found that for Chinese children in Grades 4, 5, and 6, a shift in distributive fairness occurs as they transition from late childhood to early adolescence. Both peer comparison and SVO influenced children's distributive fairness, with these effects varying across developmental stages. Notably, SVO only moderated the peer comparison effect in the DG. By comparing differences in children's distributive fairness between the DG and the UG, we further confirmed that peer comparison, SVO, and grade are critical factors influencing the variations in distributive fairness across different contexts.

### Distributive fairness among children and its developmental trends

In this study, we found that even in the DG, where children had access to all gold coins, they chose to share the benefits with the receiver, exhibiting other‐regarding preferences. Children's fair distribution trends differed between the DG and the UG. In the DG, the proportion of gold coins distributed to others remained between 30% and 40% across three grades. In contrast, in the UG, the proportion distributed by Grade 4 (49.4%) was higher than that distributed by Grade 5 (46%) and Grade 6 (45%). These results are consistent with those of previous research investigating the development of distributive fairness among Chinese children, which showed that the number distributed to others remained stable as age increased in the DG, while it gradually decreased in the UG (Zhu et al., 2008).

As individuals' distribution behavior depends on a game of fairness motivation versus social comparison motivation, we attribute the behavioral differences mentioned above to the fact that participants assigned different weights to fairness between the DG and the UG. Both tasks were conducted in a single round and anonymously, thereby giving participants absolute distributional power in the DG without fear of “revenge” from the receiver (Camerer, 2003; Hashimoto et al., 2014), which further encouraged children to maximize self‐interest via social comparison motivation. However, previous research has claimed that children establish an altruistic consciousness by age 4 (Eisenberg et al., 2016) and exhibit stable other‐regarding preferences by age 9 (Güroğlu et al., 2009). Almås et al. (2010) further revealed that although fairness consciousness increases with age as children enter adolescence, their self‐interest levels remain steady. These findings provide compelling evidence for explaining why children in the DG proposed similar altruistic unfair distributions across all grades.

In contrast, children faced declining distributive power and the risk of losing all gold coins if the receiver rejected their distributions in the UG, thereby driving all children to strategically integrate self‐interest, the needs of others, and fairness norms before making their own distribution (Steinbeis et al., 2012). At that time, fairness motivation prevailed, leading children to increase the number of distributed coins to satisfy the receiver's minimum acceptable offer (Cochard et al., 2021). It should be mentioned that only fourth graders proposed nearly equal distribution in the UG. Previous studies have revealed that younger children tend to adopt simple outcome equality as the fairness norm (Gummerum et al., 2008) and prefer to offer strategic fair distribution out of the desire for social approval (Gonzalez et al., 2021). Conversely, adolescents generally possess a higher level of moral cognition, a deeper understanding of fairness norms, and an increased sense of self‐reliance, which enables them to realize that they do not need to be overly pleasing to others (Yucel et al., 2022). Therefore, in this study, adolescents utilized distribution proposals that balanced fairness and self‐interest, whereas younger children were motivated by external incentives to propose equitable distributions. Consistent with the findings presented above, this study also observed that fourth graders had much higher difference values (Number_UG_ − Number_DG_) than upper graders. This result reconfirmed that adolescents have more consistent fair distribution criteria and more matured rational decision‐making skills than younger children.

When comparing the distribution patterns of Chinese and Western children, we found discrepancies mainly in the UG. Western children's distribution numbers increased with age, and their proposals gradually reached fair from unfair in the UG (Güroğlu et al., 2009). Different cultural backgrounds may account for this inverse tendency, as cultural values and market economic systems subconsciously shape individuals' distribution behaviors in the DG and the UG (Cochard et al., 2021). Unlike Western children, who are initially influenced by “individualism” cultural values and later develop concerns for fairness through social experiences, Chinese youngsters are imbued with “collectivism” from birth. Subsequently, they develop self‐centered behaviors under the influence of the market economy system (Zhu et al., 2008). We also compared our findings with those of previous studies conducted on adults, and found that children's distribution number in the DG was somewhat higher than those of adults (20%–30%), but equivalent in the UG (40%–50%) (Oosterbeek et al., 2004). This suggests that children aged 10–12 years can utilize strategic fairness like adults, but they may place a higher value on the interests of others and on fairness norms.

### Effect of peer comparison on distributive fairness among children

In this study, we found a gradual decrease in children's distribution number in both the DG and the UG as peers proposed increasingly unfair distributions, thereby implying that children seek conformity with their peers even when their peers engage in amoral and antisocial behavior (Ruggeri et al., 2018). Then, two critical questions must be addressed.

The first question involves establishing why distributive fairness among children is influenced by their peers. Ho and Su (2009) suggested that in the presence of peers, individuals' fairness decisions no longer rely solely on “distributional fairness concerns” between themselves and the receiver, but they significantly rely on “peer‐induced fairness concerns.” Therefore, in this study, where children were asked to evaluate peer proposals before distributing coins, they appeared to be more concerned with differences between themselves and their peers. Once children realize that their distribution patterns differ from those of their peers, they adjust their behaviors to pursue peers. The second question concerns why children prefer to align distributional tendencies with their peers. Researchers who support the social preference model have argued that individuals have a strong aversion to being different from their peers and therefore seek to achieve equal benefits with them (Gächter et al., 2013). However, this preference does not account for individuals' conformity in all contexts (Thöni & Gächter, 2015). Fabbri and Carbonara (2017) further suggested that children adhere to their peers because they view peer behavior as a social norm that must be followed. Under the influence of this social norm model, which emphasizes peer pressure, children in this study conformed to acquire group acceptance. Unlike the two preceding models that highlight the importance of peers, Cason and Mui (1998) argued that peer behavior serves merely as informative knowledge, assisting individuals in making rapid and optimum decisions in uncertain situations. From this perspective, children in this study processed peer proposals as informative guidance because they were unsure of the most reasonable distribution proposal at that moment. Although multiple viewpoints can currently explain the peer comparison effect, further studies are needed to determine which is most crucial.

Interestingly, the present study found that the direction of the peer comparison effect shifted significantly as children transitioned from late childhood to early adolescence. As the unfairness of peer distribution proposals increased, the number of distributions by fourth graders remained steady in both the DG and the UG. In contrast, the number of distributions in the higher grades gradually decreased, thereby confirming that adolescents are more susceptible to peer influence than younger children (Ruggeri et al., 2018). Changes in adolescents' internal and external environments may explain the discrepancies between these two developmental stages. On the one hand, the cognitive level of adolescents improves significantly due to the integrated and specialized nature of brain structures and functions, particularly within the prefrontal cortex (van der Aar et al., 2019). First, teenagers' meta‐cognition migrates from the “subjective self” to the “social self,” leading them to realize that the self is part of a broader social interaction (Krettenauer et al., 2019). Second, adolescents' theory of mind level has developed to the point where they can identify the intentions of others and experience others' negative emotions caused by unfairness, which inhibits antisocial motivation (Weimer et al., 2017). Third, adolescents' fairness perceptions have evolved to the point where they no longer consider outcome equality as the norm of fairness; instead, they adopt strategic fair distribution (Gonzalez et al., 2021). In this study, compared with younger children, adolescents' improved capacities for meta‐cognition, other‐related cognition, and fairness cognition enable them to balance self‐interest, the receivers' needs, and peers' distributional intentions before making proposals. On the other hand, the focus of adolescents' interpersonal relationship shifts from the family to the peer group, making peer acceptance the primary motivator of their behaviors (Fuhrmann et al., 2015). Consequently, adolescents considered their peers to be the reference standard, while younger children chose adults with expertise and extensive social experience. Consistent with previous studies, the results of this study showed that fourth graders in the late childhood stage adopt comparable distribution schemes as adults, whereas upper graders in the early adolescence stage modify their distribution behavior to conform to their peers.

Another interesting finding is that the number of distributions made by fourth graders in the DG remained steady at approximately three, even under the influence of peer comparison, whereas it gradually decreased from nearly equal to around three for upper graders. This phenomenon reaffirms that the level of individual self‐interest remains consistent from childhood to adolescence (Almås et al., 2010). Even when children are free to distribute, the most immoral proposal they tend to make is only mildly unfair, despite peers setting a bad model. In contrast to the DG, as peer proposals became increasingly unfair in the UG, the number of distributions by fourth graders remained approximately five, whereas the number distributed by fifth and sixth graders decreased from five to four, thereby confirming that younger children prefer simple equal outcomes, whereas adolescents prefer strategic fair distributions.

Furthermore, results from the difference values between the DG and the UG revealed that the largest distribution discrepancies occurred when participants viewed extremely unfair peer distribution across all three peer comparison conditions. This indicates that distributive fairness among children is context‐dependent and that at least some children chose to follow immoral but self‐interest‐maximizing proposals.

### Moderating role of social value orientation in different tasks

In this study, we found that, among Chinese primary school children, the proportion of prosocials increased as children transited to early adolescence, thereby suggesting that some prosocials adopted self‐interested distribution proposals during late childhood. The stage‐based expression of SVO can be attributed to two aspects. On the one hand, as children's moral reasoning ability improves, their perception of fairness norms extends and they become more aware of the importance of upholding these norms. On the other hand, social education and practical experience continuously emphasize the effectiveness of prosociality among children (Li et al., 2013). Given that previous studies are cross‐sectional, this characteristic of SVO should be interpreted with caution and verified through longitudinal follow‐up studies.

Except for reconfirming that prosocials distribute more resources to the receiver than proselfs (Bono et al., 2020; Pletzer et al., 2018), this study found that children with different SVOs exhibited distinct distribution patterns between the two tasks. In the DG, which measures “true fairness,” individuals distribute in accordance with their actual inner motivations. Therefore, the proselfs in this study made proposals to maximize self‐interests, whereas the prosocials made proposals for mutual interests, particularly aiming for equal benefit of both parties (Zhang et al., 2017). However, in the UG, which measures “strategic fairness,” we found that children with both SVOs made near‐fair distribution proposals. Although the prosocials and proselfs exhibited similar distributive behaviors, their underlying concerns about fairness may differ. Prosocials have a stable aversion to unfairness, and thus, they do not alter their primary goal of fair distribution regardless of the changing contexts (Heilman & Kusev, 2020). Conversely, the proselfs view strategic fairness as a solution to avoid rejection by the receiver (van Dijk et al., 2004) or to maintain good social impressions (Leimgruber et al., 2012). In other words, the findings of this study support the assumption that prosocial children are actual defenders of fairness, whereas proself children are defenders of strategic fairness.

More importantly, in this study, we found that SVO moderated the peer comparison effect in the DG. For the proselfs, as peers made increasingly unfair proposals, fourth graders adopted self‐interested, mildly unfair distributions nearly independently of peer influence, whereas the distribution criteria for fifth graders and sixth graders were gradually reduced to mildly unfair. Instead, for the prosocials, distribution trends among children were identical across grades, whereby they chose fair distribution after their peers distributed fairly. Afterward, despite reducing the number of distributions under the impact of peer comparison, all distribution proposals remained above the level of being mildly unfair. Two critical messages were sent out. First, although children were more influenced by the peers as they entered early adolescence, neither prosocials nor proselfs rely on peer distribution criteria entirely. Additionally, the proselfs' behavior was more vulnerable to peer comparison than that of the prosocials. The results of this study are similar to those of previous studies, which revealed that prosocials only follow prosocial behaviors of group members, but proselfs consistently conform to peers in the group (Wei et al., 2016). Wei et al. (2016) offered a reasonable explanation for this phenomenon, stating that prosocials strictly adhere to social norms, and therefore, when peers' immoral behavior strikes the fairness beliefs they uphold, prosocials know that their peers' behavior will not be socially approved, and thus they cling to their original fairness norms and exhibit stable prosocial behavior. Rather, proselfs put self‐interest first, and therefore, once peers' immoral behavior can result in increased benefits for themselves, they will not hesitate to seize the opportunity. This difference between the two SVOs was also reflected in the results of the difference values between the DG and the UG, where the difference values were significantly larger for the proselfs than for the prosocials, indicating that the fairness concern of prosocial children was highly stable across contexts, whereas proself children increasingly pursued self‐interest.

In this study, we did not find a moderating effect of SVO in the UG, as both prosocial and proself children consistently proposed nearly equal distributions. The discrepancy between the two experiments can be attributed to the nature of the UG, which diminishes children's distribution power and increases the risk of losing resources. This compels all children to distribute gold coins in a manner that satisfies both social norms and the expectations of others.

### Limitations and directions for future research

This study has some limitations. First, the statistically significant results in this study all had small effect sizes. A similar phenomenon was observed in the study conducted by Allgaier et al. (2020), who attributed that phenomenon to sample heterogeneity or small sample size. We then inspected the number of distributions per participant and discovered that children might make different or even opposing distributions for the same SVO, thereby implying that SVO is not the only factor at play. Some studies in recent years have zeroed their attention on the impact of social comparison orientation on fairness‐related decisions, whereby the stronger the social comparison orientation, the more susceptible an individual's behavior and emotions are to the impact of comparative information (Merriman et al., 2020). Future studies can explore whether and how social comparison orientation influences distributive fairness among children more deeply. In addition, only two of the most representative types of SVO, prosocial and proself, were selected for this study (Hu & Mai, 2021; Li et al., 2013). Considering that previous researchers have categorized SVO into three or even more types based on their different classification criteria (Murphy & Ackermann, 2014; van Lange et al., 1997), future studies cannot ignore the role of other atypical types of SVO.

Second, we have only briefly compared the distributional behaviors of children in this study with that of Western children or adults in previous research in Section 4, which is insufficient to conclude the influence of cultural factors on children's fairness behavior. Existing evidence indicates that cultural differences not only affect children's perceptions of peer relations (Engelmann et al., 2021) but may also indirectly alter prosocial behavior among children by influencing their level of moral development (Chaparro et al., 2013). Cross‐cultural studies are required in the future to bridge the existing research gap.

Finally, although this study confirms that distributive fairness among children is indeed influenced by peers, it ignores the social distance between children and their peers. Previous studies have shown that participants evaluate the behavioral outcome of their friends and strangers differently, and they desire the same benefits from their friends (Liu et al., 2021). Does this imply that the influence of friends is more powerful than that of strangers? Comparing the two types of peer influence is an important next step.

## CONCLUSION

This study confirmed significant changes in distributive fairness among children as they transitioned from late childhood to early adolescence. In both the DG and the UG, SVO and peer comparison significantly influenced children's distribution behaviors at these two developmental stages. However, SVO only moderated the peer comparison effect in the DG. These findings suggest that early adolescence may be a pivotal turning point in the development of children's fair distribution behaviors. Further research is needed to validate this conclusion.

## CONFLICT OF INTEREST STATEMENT

We have no conflicts of interest to disclose.

## ETHICS STATEMENT

This study was approved by the Ethics Committee of the Department of Psychology at Renmin University of China.

## Supporting information


**Data S1.** Supporting information.

**Figure S1.** The procedure of the first round of the DG.

**Figure S2.** The procedure of the first round of the UG.
