# Supplementary material for: Distributive fairness during the transition to adolescence: The role of peer comparison and social value orientation
Source: Psych J. 2024 Sep 18;14(1):118–30. doi: 10.1002/pchj.800 (PMC11787880; doi:10.1002/pchj.800)
Supplement: Supplementary file 1 — Data S1. Supporting information. [file PCHJ-14-118-s001.docx]

**Supplementary Materials**

**Distributive fairness during the transition to adolescence: the role of peer comparison and social value orientation**

**Contents：**

This file includes details of statistical results:

**1 Details of the experimental procedure**

- 1. Details of the procedure for DG (Task 1)
  2. Details of the procedure for UG (Task 2)

1. **Supplementary results of three-factors repeated measure ANOVA**
   1. the main effect of peer comparison
   2. the main effect of SVO
   3. the interaction of SVO × Grade × Peer comparison
2. **Exploratory analysis with Sex as a independent variable**
3. **Comparing differences between DG and UG using raw data**

**Tables S1**

**Figure S1 – S2**

**1 Details of the experimental procedure**

**1.1 Details of the procedure for the DG (Task 1)**

The participants were asked to play three rounds of the DG, and in each round the participants acted as the “distributor” and a strange student of the same grade and gender (hereinafter referred to as “Student A, B, C”) acted as the “receiver.” Figure S1 shows the first round of the DG. First, 10 virtual gold coins are presented, and the participant is informed that those are the total resources that can be distributed in each round. Afterward, images of two students are displayed. The one on the left wearing a red suit always represents the participant, whereas the one on the right wearing a blue suit represents “Student A.” The participants were told that they can distribute the 10 coins according to their real intentions, and the “Student D” can only accept the proposal without knowing who submitted it. Next, another peer distributor’s proposal was presented, thereby informing the participants that “before coming to your school, we performed the same study in another elementary school. One distributor (‘Stranger 1’) kept seven coins and gave three coins to the receiver (‘Stranger 2’), that is, 7–3.” Finally, the two students’ images and 10 coins were again presented together to the participants, and they were asked “How many coins do you plan to give Stranger A after knowing the other distributor’s proposal?” The participants were also reminded that the coins kept to themselves in each round would be added up and could be exchanged for gifts of different values, and the more the coins, the better the gift. Next, the participants completed the answer booklet without looking at other students’ choices. The participants’ selection from “0,1,2,3,4,5,6,7,8,9,10” determined the amount of gold coins distributed to the receiver. For instance, selecting “5” indicated giving five coins to “Student A” and retaining five coins for oneself.

----- INSERT FIGURE S1 ABOUT HERE -----

**1.2 Details of the procedure for the UG (Task 2)**

Task 2 comprised three rounds of the UG, during which the participants still acted as the distributor to distribute 10 coins to themselves (wearing red suit) and a different strange receiver, that us, “Student X (wearing an orange suit),” “Student Y (wearing a purple suit),” and “Student Z (wearing a pink suit).” They still viewed the three peer proposals in the same order before distribution. The most critical difference with the DG was that the participants knew that the receiver can reject their proposals, and if that happened, both parties gained zero coins (Figure 2). Including rest time, the study took 25 minutes in total.

----- INSERT FIGURE S2 ABOUT HERE -----

**2 Supplementary results of three-factors repeated measure ANOVA**

**2.1 Main effect of peer comparison**

In the DG, the main effect of peer comparison was significant, *F* (2, 1078) =7.52, *p* = .001, *η*_p_^2^ = 0.01. Post-hoc multiple comparison results showed that the more unfair the distribution proposal provided by the peer, the less the number of gold coins distributed to the receiver by the children (*M ± SD*, Fair: 4.01 ± 2.15; Mildly unfair: 3.55 ± 2.08; Extremely unfair: 3.00 ± 2.41), and *p*-values for comparison between any two conditions were less than .001.

The main effect of peer comparison was also significant in the UG, *F* (2, 1078) = 6.84, *p* = .001, *η*_p_^2^ = 0.01. Post-hoc multiple comparison results showed the same distribution trends as the DG. The number of gold coins distributed by children reduced significantly with increasing peer proposal’s unfairness, all *p ≤* .001 (*M ± SD*, Fair: 5.01 ± 1.64; Mildly unfair: 4.62 ± 1.64; Extremely unfair: 4.37 ± 1.87).

**2.2 Main effect of SVO**

In the DG, the SVO’s main effect was significant, *F* (1, 539) = 46.49, *p* < .001, *η*_p_^2^ = 0.08. The number of distributions was significantly lower for proself children (2.96 ± 1.95) than for prosocial children (4.06 ± 1.79).

In the UG, the SVO’s main effect was also significant, *F* (1, 539) = 21.84, *p* < .001, *η*_p_^2^ = 0.04. The number of gold coins distributed by the prosocials (4.95 ± 1.43) was significantly more than that distributed by the proselfs (4.39 ±1.35).

**2.3 Interaction of SVO × Grade × Peer comparison**

Unlike the DG, we did not find a three-way interaction of SVO × Grade × Peer comparison in the UG, *F* (4, 1078) = 1.57, *p* = .185, *η*_p_^2^ = 0.01. Nonetheless, it is necessary to be mindful of the considerable heterogeneity in the distribution number of children in the same grade or peer-comparison condition, which might result in the elimination of the interaction. Therefore, we conducted a comparison analysis of the number of children’s distributions across all conditions.

For fourth-grade participants, there was no significant difference between the number of gold coins distributed by the proself children in the three peer-comparison conditions (*p*s > .289), whereas the prosocial children distributed fewer gold coins in both the peer mildly unfair distribution condition (*p* = .002) and the peer extremely unfair distribution condition (*p* = .040) than in the peer fair distribution condition.

For fifth-grade participants, proself children distributed significantly fewer gold coins in the mildly unfair peer distribution (*p* = .002) and extremely unfair peer distribution (*p* < .001) conditions than in the fair peer distribution condition, but the difference between the former two conditions was not significant (*p* = .461). In contrast, prosocial children distributed considerably fewer gold coins in the peer extremely unfair distribution condition compared to the peer fair distribution and peer mildly unfair distribution conditions (*p* = .001 and *p* = .027, respectively).

As for the sixth-grade participants, the number of distributions distributed by the proselfs gradually decreased with the increase of peer distribution proposals’ unfairness, *p*s < .023. In contrast to the proselfs’ distributional pattern, the prosocials distributed significantly fewer gold coins in the peer extremely unfair distribution condition compared to the peer fair distribution condition (*p* < .001) and the peer mildly unfair distribution condition (*p* = .003), with no significant difference between the latter two conditions (*p* = .107).

**3 Exploratory analysis with Sex as a independent variable**

We performed a 2(Sex: male vs. female) × 2 (SVO: prosocial vs. proself) × 3 (Grade: Grade 4 vs. Grade 5 vs. Grade 6) × 3 (peer comparison: fair vs. mildly unfair vs. extremely unfair) repeated measures analysis of variance (rmANOVA) for both DG and UG.

In the DG, the main effect of Sex was not significant, *F*(1, 534) = 0.65, *p* = .420. The interaction effects of Sex × Peer comparison, Sex × SVO, Sex × Grade, Sex × SVO × Peer comparison, Sex × SVO × Grade, Sex × Grade × Peer comparison, Sex × SVO × Grade × Peer comparison were not significant (*p*s ≥ .321).

Consistent with the results of DG, the main effect of Sex in the UG was also not significant (*F*(1, 534) = 1.57, *p* = .210). Moreover, all interaction effects including Sex were not significant (*p*s ≥ .347).

Therefore, we believed that sex was not the main factor influencing the distribution behavior of children.

**4 Comparing differences in raw data between two tasks**

In the main text, we calculated the difference in the number of gold coins distributed between the two tasks for each participant and used this as an indicator of the difference between the tasks. In order to present a more comprehensive information about the data, we proceeded to compare the actual distributional number of participants between the two tasks. Thus, a 2 (Task type: DG vs. UG) × 2 (SVO: prosocial vs. proself) × 3 (Grade: Grade 4 vs. Grade 5 vs. Grade 6) × 3 (peer comparison: fair vs. mildly unfair vs. extremely unfair) rmANOVA was applied.

We found that the main effect of task type was significant, the number of gold coins distributed by the participants in the UG was significantly more than the number of gold coins distributed in the DG, *F* (1, 539) = 56.69, *p* < .001, *η*_p_^2^ = 0.10. The main effect of SVO was significant, prosocial participants distributed more gold coins than proself participants, *F* (1, 539) = 44.30, *p* < .001, *η*_p_^2^ = 0.08. The main effect of peer comparison was significant, *F* (2, 1078) = 12.12, *p* < .001, *η*_p_^2^ = 0.02. The number of gold coins distributed by the participants decreased significantly as the unfairness of the distribution proposal made by their peers increased, *p*s < .001.

The interaction of peer comparison and grade was significant, *F* (4, 1078) = 4.66, *p* = .001, *η*_p_^2^ = 0.02. The results of the simple effect analysis showed that, as the unfairness of the peer distributions increased, fifth- and sixth-grade children distributed significantly fewer gold coins to others (*p*s < .001). Differently, for fourth-grade children, compared to peers proposing fair distribution, the number of gold coins distributed by the participants was significantly lower when peers proposed mildly unfair distribution (*p* = .004) and extremely unfair distribution (*p* < .001), but there was no significant difference between the latter two conditions. The interaction of task type and grade was significant, *F* (2, 539) = 12.11, *p* < .001, *η*_p_^2^ = 0.04. The simple effect analysis revealed that all three grades of participants, distributed significantly more gold coins in UG than in DG, *p*s < .001. The interaction of task type and SVO was significant, *F* (2, 539) = 16.01, *p* < .001, *η*_p_^2^ = 0.03. The simple effect analysis revealed that both in DG and UG, prosocial participants distributed more gold coins than proself participants, *p*s < .001.

The interaction of peer comparison × SVO × grade was significant, *F* (4, 1078) = 3.01, *p* = .02, *η*_p_^2^ = 0.01. For fifth- and sixth-grade children, prosocials and proselfs exhibited the same behavioral tendency, i.e., the more unfair the distributions proposed by their peers, the lower the number of gold coins they actually distributed, *p*s < 0.004. For fourth-grade children, the number of gold coins distributed by proselfs did not differ as the degree of unfairness of the distributions proposed by their peers changes. However, the number of gold coins distributed by prosocials decreased as the degree of unfairness of peer distributions programs increased (*p*_(Fair vs. Mildly Unfair)_ = .039, *p*_(Fair vs. Extremely Unfair)_ < .001, *p*_(Mildly Unfair vs. Extremely Unfair)_ = .062). No other main or interaction effects were significant.

**Table S1**

*Mean and Standard Deviation of children's distribution number under three peer comparison conditions at grade 4, 5, 6*

|  |  | **Distribution numbers (*M* ± *SD*)** | | |
| --- | --- | --- | --- | --- |
|  |  | Fair | Mildly Unfair | Extremely Unfair |
| ***DG*** |  |  |  |  |
|  | Grade 4 | 3.52 ± 2.42 | 3.29 ± 2.47 | 2.91 ± 2.56 |
|  | Grade 5 | 4.40 ± 1.95 | 3.89 ± 1.75 | 3.37 ± 2.25 |
|  | Grade 6 | 4.27 ± 1.92 | 3.67 ± 1.92 | 3.11 ± 2.30 |
| ***UG*** |  |  |  |  |
|  | Grade 4 | 5.13 ± 1.73 | 4.75 ± 1.71 | 4.81 ± 1.92 |
|  | Grade 5 | 4.97 ± 1.25 | 4.63 ± 1.42 | 4.31 ± 1.85 |
|  | Grade 6 | 4.98 ± 1.50 | 4.63 ± 1.40 | 4.14 ± 1.84 |

**Figure captions**

Figure S1. The procedure of the first round of the DG.

Figure S2. The procedure of the first round of the UG.
