# Supplementary figures and images for: Distributive fairness during the transition to adolescence: The role of peer comparison and social value orientation
Source: Psych J. 2024 Sep 18;14(1):118–30. doi: 10.1002/pchj.800 (PMC11787880; doi:10.1002/pchj.800)

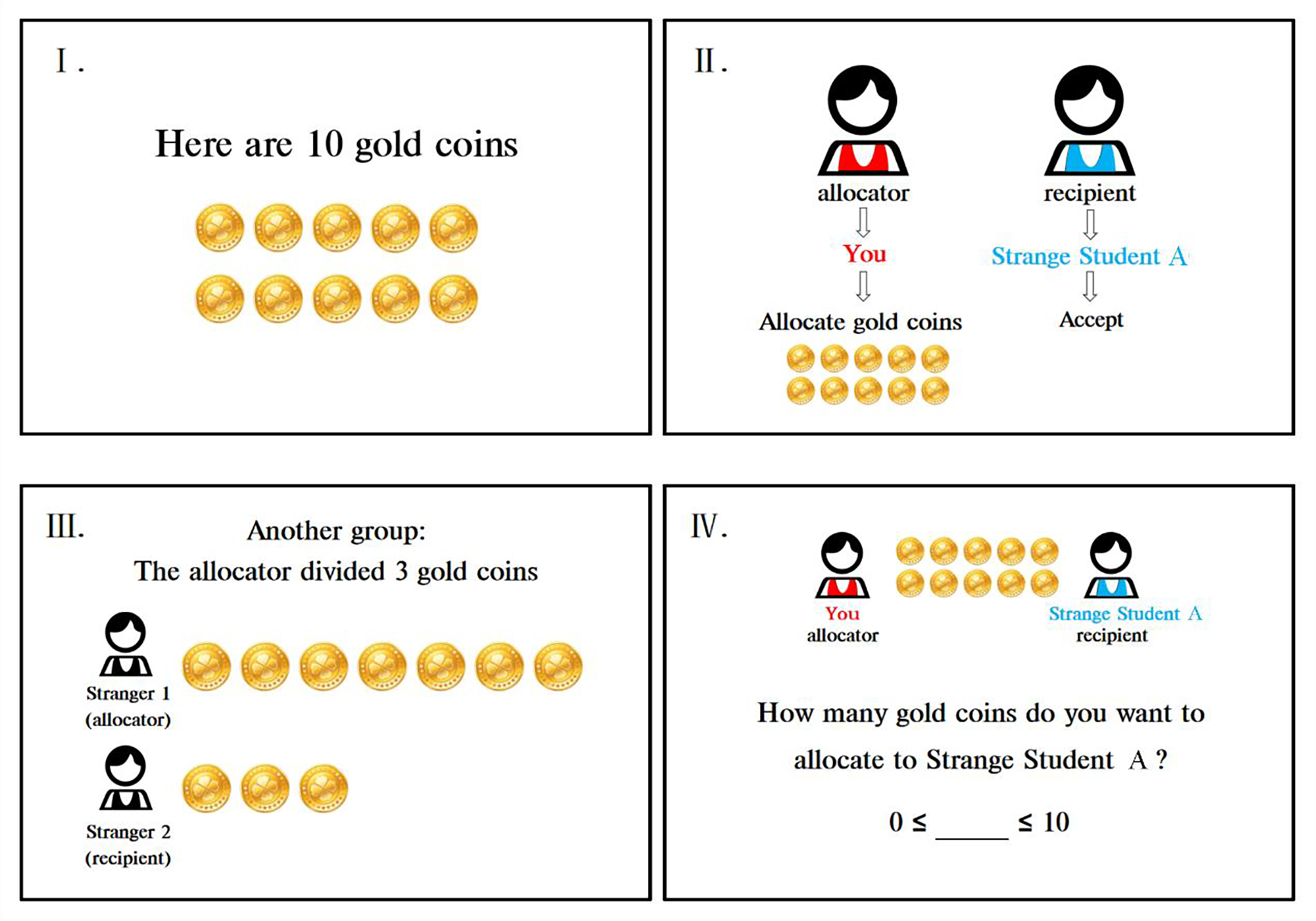

Supplement: Supplementary file 2 — Figure S1. The procedure of the first round of the DG. [file PCHJ-14-118-s002.tif]

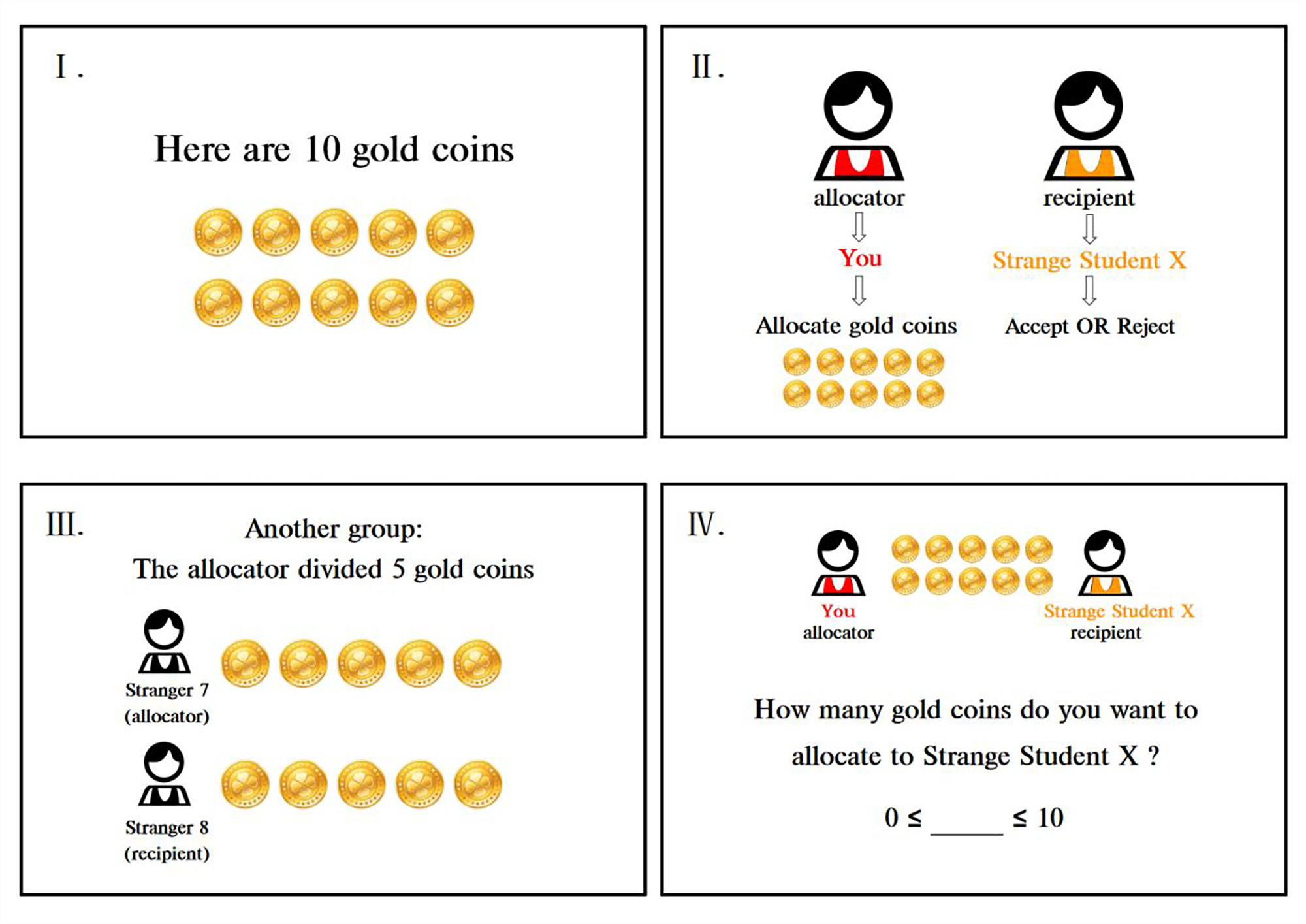

Supplement: Supplementary file 3 — Figure S2. The procedure of the first round of the UG. [file PCHJ-14-118-s003.tif]
